# Knockdown of ubiquitin-like modifier-activating enzyme 2 promotes apoptosis of clear cell renal cell carcinoma cells

**DOI:** 10.1038/s41419-021-04347-7

**Published:** 2021-11-09

**Authors:** Guoxi Zhang, Junrong Zou, Jinglin Shi, Biao Qian, Kaiyang Qiu, Quanliang Liu, Tianpeng Xie, Zhihua He, Hui Xu, Yunfeng Liao, Yuting Wu, Yanmin Li, Guancheng Xiao, Yuanhu Yuan, Rihai Xiao, Gengqing Wu, Xiaofeng Zou

**Affiliations:** 1 Department of Urology, First Affiliated Hospital of Gannan Medical University, Ganzhou, Jiangxi 341000 China; 2 Department of Urology, Wan’an People’s Hospital, Ji’an, Jiangxi 343800 China

**Keywords:** Oncogenes, Oncogenesis

## Abstract

Small ubiquitin-related modifier (SUMO) proteins are involved in the development of tumors. Ubiquitin-like modifier-activating enzyme 2 (UBA2) is an important member of the SUMO modification system; however, its role in clear cell renal cell carcinoma (ccRCC) is unclear. Therefore, we investigated the expression and function of UBA2 in ccRCC. Both mRNA and protein expression levels of UBA2 were found to be higher in ccRCC than in normal renal tissues and significantly related to the tumor size, Fuhrman grade, and tumor stage. UBA2 knockdown inhibited ccRCC cell growth, promoted apoptosis in vitro and in vivo, and decreased the abundance of a p53 mutant, c-Myc, and key enzymes of the SUMO modification system. Meanwhile, overexpression of UBA2 had the opposite effects. Overexpression of the p53 mutant or c-Myc alleviated the effects of UBA2 knockdown on ccRCC cell proliferation and apoptosis. In conclusion, targeting UBA2 may have a therapeutic potential against ccRCC.

## Introduction

Renal cell carcinoma (RCC) accounts for 80 to 90% of renal malignancies and ranks second only to bladder cancer among malignant tumors of the urinary system. RCC mainly includes clear cell RCC (ccRCC) and non-clear cell RCC, with the former accounting for ~75–85% and the latter accounting for ~15–20% of all cases. According to the 2015 cancer statistics in China, the incidence and mortality rate of RCC were 66.8 and 23.4 per 100,000, based on the average incidence rates for the most recent 3 years (2009–2011) of data from 72 population-based cancer registries [1]. Once metastasis occurs, the 5-year survival rate for patients with RCC is <20% [2]. Currently, surgery is the main treatment for RCC. For metastatic RCC, more comprehensive treatment options have been adopted, especially with the emergence of molecular targeted therapy drugs in recent years, bringing a new dawn to the treatment of RCC. However, the prospects regarding the efficacy of existing RCC drugs are still not very optimistic, and the main reasons are that the pathogenesis of renal cancer is not fully understood yet and there is a lack of drugs with strong targets. Therefore, it is imperative to further elucidate the pathogenesis of RCC and develop new anti-RCC drugs.

Small ubiquitin-related modifiers (SUMOs) are a highly conserved protein family, which is widely found in eukaryotes [3, 4]. SUMO modification competitively blocks ubiquitin binding, thereby blocking the degradation of substrate proteins by the ubiquitin–proteasome system, a crucial protein degradation system in eukaryotes [3, 4]. Studies increasingly suggest that SUMO modification is involved in the occurrence and development of tumors [5, 6]. Ubiquitin-like modifier-activating enzyme 2 (UBA2), also known as SUMO-activating enzyme subunit 2 (SAE2), forms a heterodimer E1 ligase with SAE1. In addition to being involved in SUMOylation and ubiquitin-mediated proteolysis, UBA2 plays role in regulating cancer cell proliferation, apoptosis, and metastasis in lung and colorectal cancers [7–9]. These findings suggest that UBA2 may be a target for tumor therapy. However, its role in RCC is completely unknown.

The effects of drug therapies on malignant tumors are largely dependent on apoptosis, a programmed process of cell death, which is controlled by specific genes and plays an important role in maintaining homeostasis, organ formation, and body development [10, 11]. It is of great significance to find more targeted molecules that can effectively promote the apoptosis of tumor cells or enhance the proapoptotic effects of antitumor drugs. In this study, we aimed to investigate the expression and function of UBA2 in ccRCC, with a special focus on the role of UBA2 in regulating RCC apoptosis, as well as on the underlying mechanisms.

## Materials and methods

### Clinical specimens

Human ccRCC tissues and adjacent normal renal (NR) tissues were obtained at the First Affiliated Hospital of Gannan Medical University from July 2016 to July 2017. All patients enrolled in this study had complete clinical data, including the sex, age, tumor size, Fuhrman grade, and tumor stage. A total of 11 female and 19 male patients were enrolled, with their ages ranging from 20 to 82 years. All patients understood the purpose of this study and signed an informed consent form. This study was approved by the Committee of Ethics of Gannan Medical University in Ganzhou city according to the guidelines established in the Declaration of Helsinki.

### Immunohistochemistry (IHC)

All tissues were fixed in a 10% formaldehyde solution, then embedded in paraffin, and sliced into 5-μm sections. The tissue slides were deparaffinized, rehydrated, and then retrieved with citric acid buffer (pH 6.0, 10 mM) using a standard microwave-based method. Thereafter, the sections were washed and incubated with a blocking solution for 1 h at room temperature after treated with 3% hydrogen peroxide for 10 min. Afterward, the sections were incubated with diluted primary antibodies at 4 °C overnight, followed by washing and incubation with a horseradish peroxidase (HRP)-conjugated secondary antibody. After 30 min at 37 °C, a 3,3′-diaminobenzidine solution was added for color development, and then the sections were counterstained with hematoxylin, dehydrated, and mounted. The results of staining were assessed by two independent pathologists.

All primary antibodies were purchased from Abcam (Cambridge, MA, USA), including anti-UBA2 (ab185955, 1:100), anti-mutant p53 (ab32049, 1:50), anti-c-Myc (ab32072, 1:100), anti-Bax (ab32530, 1:200), anti-cleaved caspase-3 (ab32042, 1:300), and anti-cleaved caspase-9 (ab2324, 1:100).

### Cell line

The 786-O ccRCC cell line was purchased from the Cell Bank of the Chinese Academy of Sciences (Shanghai, China). According to the information from Cellosaurus (https://web.expasy.org/cellosaurus/), 786-O cell line has a missense mutation, R248W (CGG → TGG), in codon 248 of exon 7 of the p53 gene. We also further verified through sequencing.

### Construction of stable UBA2 knockdown (KD) and overexpression (OE) cells

The full-length *UBA2* coding sequence was cloned into pLVX-ZsGreen-Puro, and a short hairpin RNA targeting *UBA2* (shUBA2; GAGTAGATTTGATATCAAA) was cloned into pLVX-Puro. Subsequently, 786-O cells were infected with packaged lentiviruses expressing UBA2 or shUBA2 to construct stable UBA2 OE and KD cells, respectively, or with empty lentiviruses as a negative control (NC).

### Experimental design

To investigate the role of UBA2 in vitro and in vivo, four groups of 786-O cells, including non-transfected (cell), NC, UBA2-KD, and UBA2-OE cells, were used. To investigate the roles of the p53 mutant and c-Myc in UBA2-mediated apoptosis, an empty pcDNA3.1 plasmid (EM), a mutant p53 overexpression plasmid (mp53-OE), and a c-Myc overexpression plasmid (c-Myc-OE) were transfected into NC and UBA2-KD cells.

### RNA extraction and quantitative reverse transcription–polymerase chain reaction (qRT-PCR) analysis

RNA extraction, qRT-PCR, and the calculation of relative mRNA expression levels were performed as described in our previous study [12]. The forward and reverse primer sequences for UBA2 were 5′-CCAAGAAAGAAGCTTCTTGT-3′ and 5′-TATCTTGTCTTGTAAGGTGA-3′, respectively.

### Western blotting

Total protein was isolated from cells or tissues using RIPA buffer, consisting of PMSF (1 mM), Aprotinin (2 µg/mL), Leupeptin (5 µg/mL), Pepstatin A (1 µg/mL), EDTA (5 mM), EGTA (1 mM). After quantification of the protein concentrations, 30 μg of total protein was separated by sodium dodecyl sulfate polyacrylamide gel electrophoresis, and the separated proteins were transferred to a polyvinylidene difluoride membrane using semi-dry transfer method. The membrane was blocked with 5% no-fat milk, and incubated with primary antibodies at 4 °C overnight, followed by incubation with HRP-conjugated secondary antibodies. The blots were stained with Immobilon western chemiluminescent HRP substrate (Millipore, Billerica, MA, USA) in the dark and then exposed to an X-ray film. All primary antibodies were purchased from Abcam, including anti-UBA2 (ab185955, 1:1000), anti-mutant p53 (ab32049, 1:1000), anti-c-Myc (ab32072, 1:1000), anti-Bax (ab32530, 1:2000), anti-cleaved caspase-3 (ab32042, 1:5000), anti-cleaved caspase-9 (ab2324, 1:1000), anti-UBC9 (ab75854, 1:1000), anti-PIAS1 (ab109388, 1:2000), anti-SENP1 (ab108981, 1:2000), and anti-GAPDH (ab205718, 5000), which was used as a loading control. According to the method described by Guo et al. [13], IOD of each band was measured and relative protein level was calculated using the ratio of the target protein to GAPDH. The relative protein level in the control group was normalized as 1.

### Cell proliferation assay

The CellTiter 96^®^ AQ_ueous_ One Solution cell proliferation assay kit (Promega, Madison, WI, USA) was used to measure cell proliferation. Briefly, after cells were cultured in 96-well plates for 24, 48, and 72 h, the CellTiter 96 AQ_ueous_ One Solution reagent was added to each well, and the plates were incubated at 37 °C, 5% CO_2_ for an additional 4 h. Finally, the absorbance was measured at 490 nm using a Multiskan MK3 microplate reader (Thermo Fisher Scientific, Waltham, MA, USA).

### Annexin V-fluorescein isothiocyanate (FITC)/propidium iodide (PI) apoptosis assay and PI staining

An annexin V-FITC/PI apoptosis assay kit (Keygen, Nanjing, China) was used to evaluate the extent of cell apoptosis. Briefly, cells were cultured in a 6-well plate for 48 h, then harvested, and resuspended in 1× binding buffer. A cell suspension (0.5 mL; 5 × 10^5^ cells) was transferred to a clean centrifuge tube and incubated with 1.25 μL of annexin V-FITC in the dark for 15 min at room temperature (18–24 °C). After centrifugation at 1000 × *g* for 5 min to remove the supernatant, the cell pellet was gently resuspended in 0.5 mL of precooled 1× binding buffer, and 10 μL of PI was added to the cell suspension. Finally, the rate of apoptosis was analyzed using a BD Accuri C6 flow cytometer (BD Biosciences, Bedford, MA, USA). Data were analyzed using BD Accuri C6 Plus Software version 1.0.23.1 (BD Biosciences, Bedford, MA, USA).

PI staining was performed using a PI solution (Solarbio Science & Technology Co., Ltd., Beijing, China) to determine the level of cell apoptosis.

### Subcutaneous tumor formation in nude mice

Cell, NC, UBA2-KD, and UBA2-OE 786-O cells (5 × 10^6^) were subcutaneously injected into BALB/c nude mice (6-week old, *n* = 6 per group), and the length and width of subcutaneous tumors were measured. The tumor volume was calculated using the following formula: (length × width^2^)/2. The tumor growth curve was drawn based on the tumor volume. After 28 days, the nude mice were euthanized by intraperitoneal injection of 130 mg/kg pentobarbital, and subcutaneous tumors were isolated and photographed. Subsequently, the tumors were fixed in a 10% formaldehyde solution and embedded in paraffin for IHC analysis. All experimental procedures were performed in accordance with the Guide for the Care and Use of Laboratory Animals (NIH publication no. 80–23, revised 1996) and approved by the Committee of Ethics of Gannan Medical University in Ganzhou city. No randomization was used and no blinding was done in this experiment.

### Bioinformatic analysis

UBA2 mRNA expression levels in kidney renal clear cell carcinoma (KIRC) was analyzed using the Gene Expression Profiling Interactive Analysis (GEPIA) database, a web server based on the data from The Cancer Genome Atlas and The Genotype-Tissue Expression [14].

### Statistical analysis

Statistical analysis was performed using the SPSS 19.0 software (IBM, Chicago, IL, USA). The results are shown as the mean ± standard deviation. Except for the data of UBA2 mRNA expression in clinical specimens, other data were normally distributed. Fisher’s exact test was used to analyze the correlations between UBA2 protein levels and clinicopathological characteristics of patients with ccRCC. Statistical comparison between two groups was performed using a *t*-test (normal distribution) or Mann–Whitney *U* tests (non-normal distribution), and that among more than two groups was carried out using one-way analysis of variance, followed by a post hoc Tukey’s test. A value of *p* < 0.05 was considered statistically significant.

## Results

### UBA2 levels in clinical specimens and their correlation with clinicopathological characteristics of patients

**Fig. 1:**
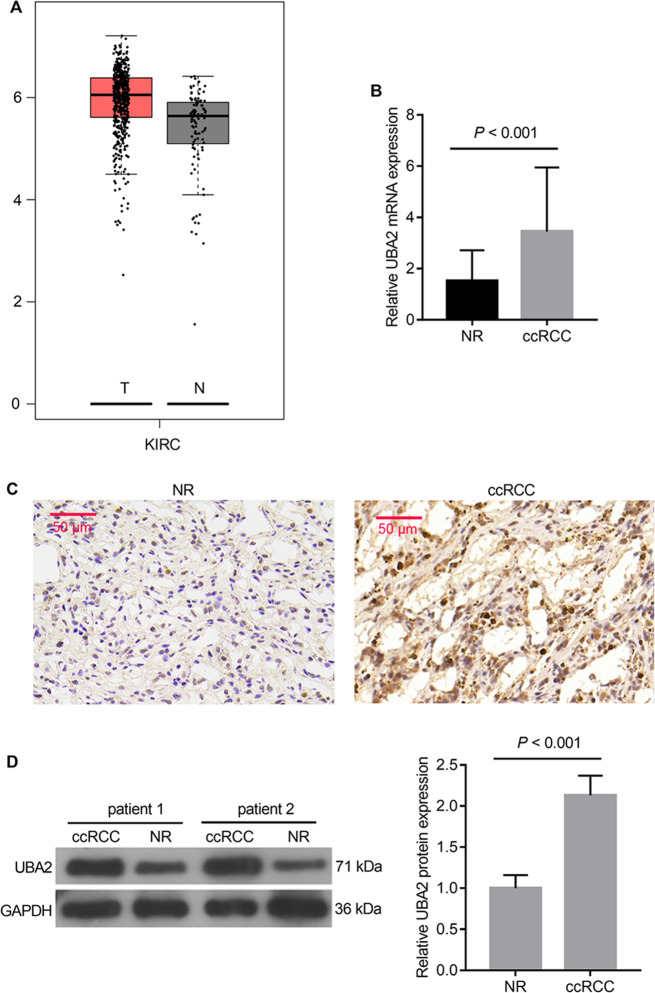
UBA2 levels in ccRCC tissues. **A** *UBA2* mRNA expression levels in the Gene Expression Profiling Interactive Analysis database. The red bar indicates kidney renal clear cell carcinoma (KIRC) tumor tissue (*T*, *n* = 523), and the gray bar indicates normal kidney tissue (*N*, *n* = 100). (**B**) *UBA2* mRNA expression levels in 30 pairs of ccRCC and adjacent NR tissues from patients. **C** Immunohistochemical staining of ccRCC and adjacent NR tissues for UBA2 (100×). **D** Western blot analysis of UBA2 levels in ccRCC and adjacent NR tissues. ccRCC clear cell renal cell carcinoma, NR normal renal tissue.

The analysis from GEPIA database showed that the *UBA2* mRNA expression levels were higher in tumor tissues than in normal tissues (Fig. 1A). Thereafter, the UBA2 levels were determined in the 30 pairs of ccRCC and adjacent NR tissues from patients. The results showed that both mRNA and protein levels of UBA2 were higher in ccRCC tissues than in NR tissues (Fig. 1B–D).

As shown in Table 1, the UBA2 protein expression levels were not related to the age and sex of the patients but were significantly related to the tumor size, Fuhrman grade, and tumor stage.

**Table 1:** Correlation of UBA2 protein levels with demographic and clinicopathological characteristics of patients with clear cell renal cell carcinoma.

Characteristics	*n* = 30	UBA2 expression level		*p*-value
		High	Low	
Sex				
Male	19	13	6	0.6956
Female	11	6	5	
Age (years)				
>57	15	8	7	0.4497
≤57	15	11	4	
Tumor size (cm)				
≤7	13	5	8	0.0227
>7	17	14	3	
Fuhrman grade				
G_1+2_	13	4	9	0.0021
G_3+4_	17	15	2	
Tumor stage				
I + II	12	3	8	0.0045
III + IV	18	16	3	

### Effects of UBA2 on cell proliferation and apoptosis in vitro

**Fig. 2:**
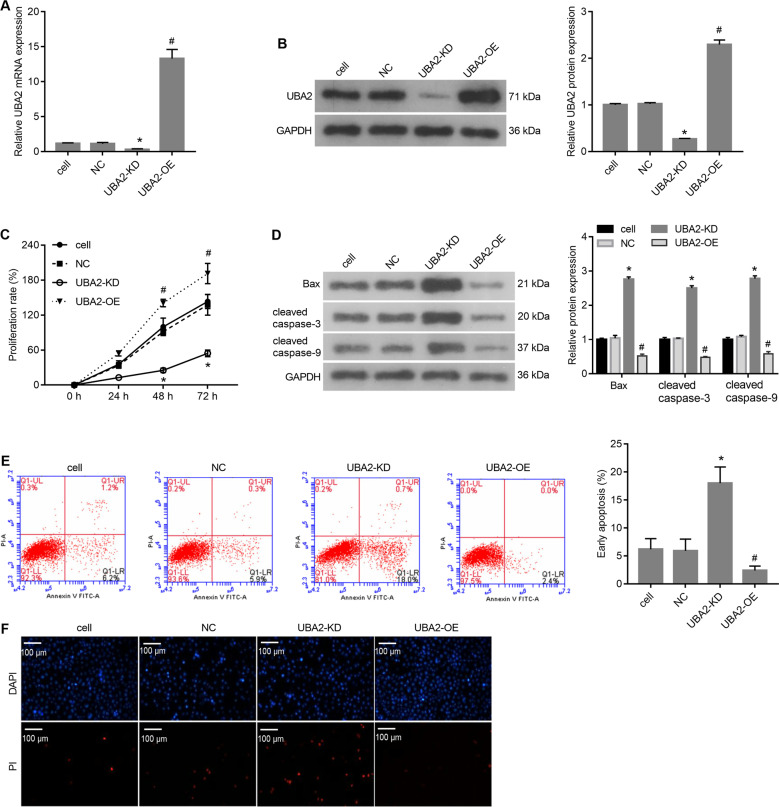
Effects of UBA2 expression levels on proliferation and apoptosis of 786-O cells in vitro. **A** mRNA and **B** protein expression levels of UAB2 in four groups of cells. **C** Rates of the proliferation of four groups of cells. **D** Expression levels of proapoptotic proteins in four groups of cells. **E** Percentages of apoptotic cells measured using the annexin V-FITC/PI assay. **F** PI staining of four groups of cells. *p < 0.05 UAB2-KD vs. NC; ^#^*p* < 0.05 UAB2-OE vs. NC. OE overexpression, KD knockdown, NC negative control, DAPI 4′,6-diamidino-2-phenylindole, PI propidium iodide.

The qRT-PCR and western blotting results showed that UBA2 was distinctly upregulated in UAB2-OE cells and distinctly downregulated in UAB2-KD cells compared with cell and NC cells (Fig. 2A, B). Therefore, it was concluded that UAB2-KD and UAB2-OE cells were suitable for use in loss-of-function and gain-of-function assays. Next, we investigated the effects of UBA2 on cell proliferation and apoptosis in vitro using cell, NC, UAB2-KD, and UAB2-OE cells. The results showed that the proliferation rate of UAB2-KD cells was significantly lower, while that of UAB2-OE cells was significantly higher than that of NC cells after culture for 48 and 72 h (Fig. 2C). In addition, the levels of the proapoptotic proteins Bax, cleaved caspase-3, and cleaved caspase-9 were obviously higher in UAB2-KD cells and lower in UAB2-OE cells than in NC cells (Fig. 2D). The results of the annexin V-FITC/PI apoptosis assay and PI staining showed that the proportion of apoptotic cells was significantly higher in the UAB2-KD cell population and significantly lower in the UAB2-OE cell population than in that of NC cells (Fig. 2E, F). These results indicated that UBA2 knockdown inhibited cell proliferation and promoted apoptosis, while UBA2 overexpression promoted cell proliferation and suppressed apoptosis in vitro.

### Effects of UBA2 on the expression of the p53 mutant, c-Myc, and key enzymes of the SUMO modification system

**Fig. 3:**
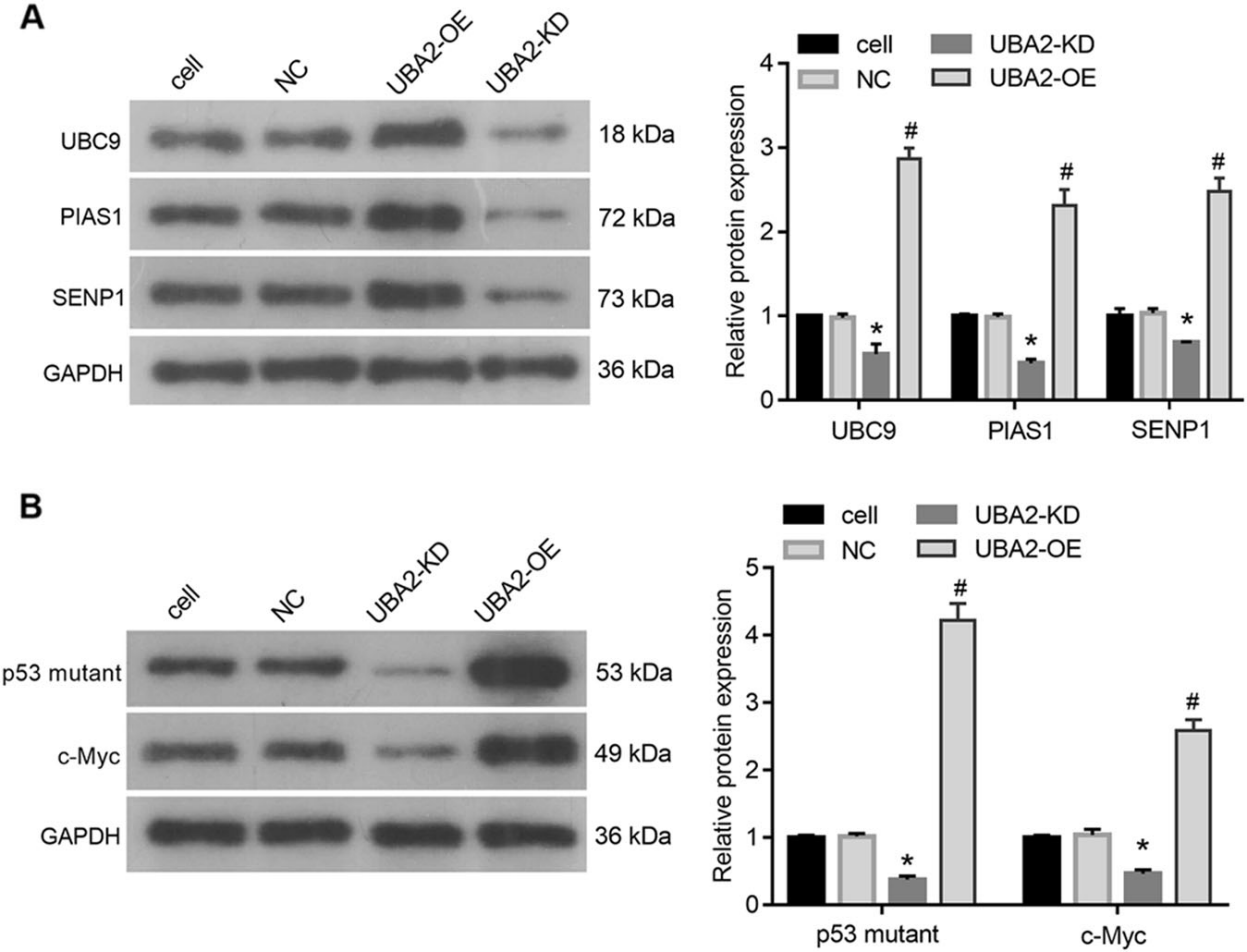
Effects of UBA2 expression levels in 786-O cells on those of the p53 mutant, c-Myc, and key enzymes of the SUMO modification system. **A** Expression levels of the key enzymes of the SUMO modification system in four groups of cells. **B** Expression levels of the p53 mutant and c-Myc in four groups of cells. OE overexpression, KD knockdown, NC negative control.

To explore the molecular mechanism of the UBA2 effects on cell proliferation and apoptosis, we analyzed the levels of p53 mutant, c-Myc, and key enzymes of the SUMO modification system. As shown in Fig. 3A, the levels of UBC9, PIAS1, and SENP1, which are the key enzymes of the SUMO modification system, were obviously lower in UAB2-KD cells and higher in UAB2-OE cells compared with those in NC cells. Moreover, the levels of the p53 mutant and c-Myc were obviously lower in UAB2-KD cells and higher in UAB2-OE cells compared with those in NC cells (Fig. 3B).

### Effects of UBA2 on tumor growth and apoptosis in vivo

**Fig. 4:**
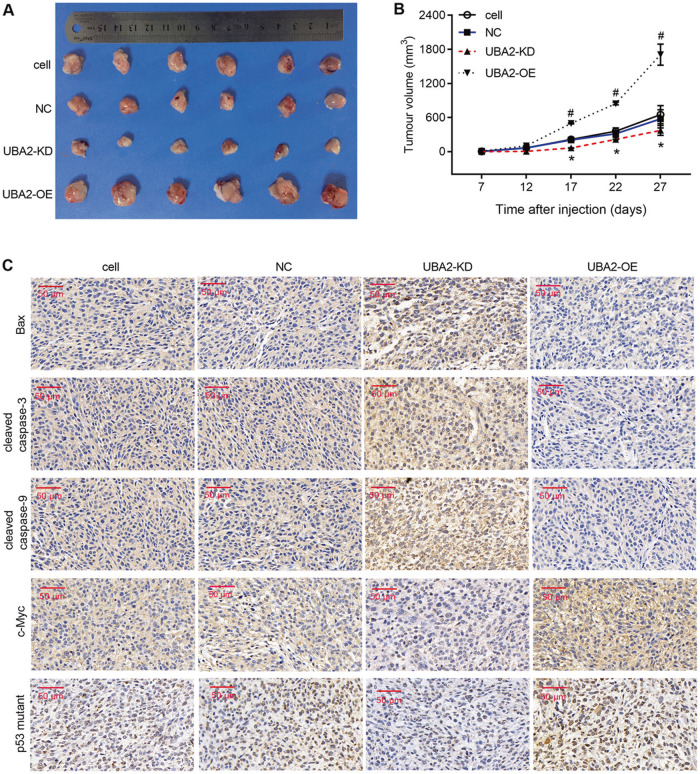
Effects of UBA2 expression levels on tumor growth and apoptosis in a nude mouse model of subcutaneous tumor formation. **A** Comparison of subcutaneous tumors induced in mice by subcutaneous injection of 786-O cells from four groups. **B** Time course of tumor growth in mice injected with 786-O cells from four groups. **C** Immunohistochemical detection of Bax, cleaved caspase-3, cleaved caspase-9, p53 mutant, and c-Myc expression in subcutaneous tumors of mice injected with 786-O cells from four groups. *p < .05 UAB2-KD vs. NC; ^#^*p* < .05 UAB2-OE vs. NC. OE overexpression, KD knockdown, NC negative control.

To further verify the role and underlying mechanism of UBA2 in ccRCC, we constructed a nude mouse model of subcutaneous tumor formation. The tumor sizes were measured at 7, 12, 17, 22, and 27 days after injection of 786-O cells to mice. As shown in Fig. 4A, B, the tumor sizes were distinctly larger in the UAB2-OE group and distinctly smaller in the UAB2-KD group compared with those in the cell and NC groups. In addition, the levels of Bax, cleaved caspase-3, and cleaved caspase-9 were obviously higher in subcutaneous tumors generated by UAB2-KD cells and lower in tumors generated by UAB2-OE cells than in those generated by NC cells. The levels of the p53 mutant and c-Myc were obviously lower in subcutaneous tumors generated by UAB2-KD cells and higher in tumors generated by UAB2-OE cells than in those generated by NC cells. Thus, UBA2 knockdown inhibited the tumor growth and promoted apoptosis, while UBA2 overexpression promoted the tumor growth and suppressed apoptosis in vivo.

### Overexpression of the p53 mutant alleviated the effects of UBA2 knockdown on cell proliferation and apoptosis

**Fig. 5:**
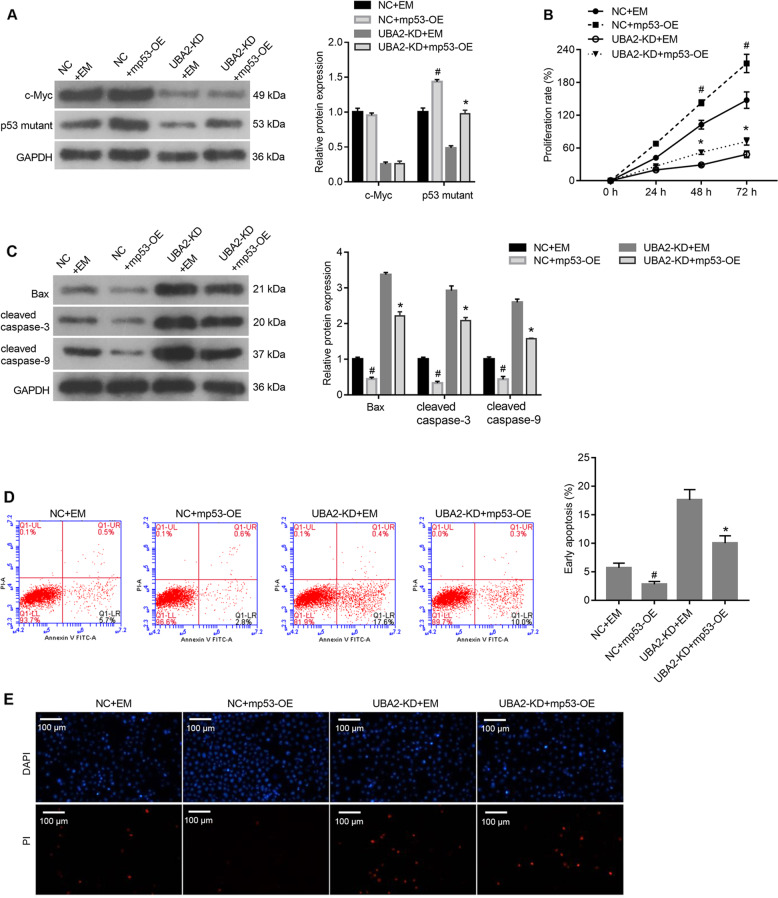
Alleviation of the effects of UBA2 knockdown on 786-O cell proliferation and apoptosis via overexpression of the p53 mutant. **A** Expression levels of the p53 mutant and c-Myc in cells transfected with mp53-overexpressing and control plasmids. **B** Proliferation rates of cells in (**A**). **C** Expression levels of proapoptotic proteins in cells in (**A**). **D** Proportions of apoptotic cells in the populations of cells in (**A**). **E** PI staining of cells in (**A**). ^#^*p* < 0.05 NC + mp53-OE vs. NC + EM; *p < 0.05 UBA2-KD + mp53-OE vs. UBA2-KD + EM. DAPI 4′,6-diamidino-2-phenylindole, PI propidium iodide, OE overexpression, KD knockdown, NC negative control, mp53 p53 mutant, EM empty vector.

To examine whether UBA2 exerts its effects via a mechanism involving the p53 mutant, the mp53-OE plasmid was transfected into NC and UBA2-KD 786-O cells. NC and UBA2-KD cells transfected with the EM plasmid were used as controls. As shown in Fig. 5A, the expression level of the p53 mutant was obviously higher in NC + mp53-OE cells than in NC + EM cells and in UBA2-KD + mp53-OE cells than in UBA2-KD + EM cells. The expression level of the c-Myc had no change between NC + mp53-OE cells and NC + EM cells or between UBA2-KD + mp53-OE cells and UBA2-KD + EM cells. These results indicate that the mp53-OE plasmid successfully overexpressed the p53 mutant, without affecting the c-Myc expression levels in the cells.

Next, we evaluated whether overexpression of the p53 mutant alleviates the effects of UBA2 knockdown on cell proliferation and apoptosis. As shown in Fig. 5B, the proliferation rate of NC + mp53-OE cells was higher than that of NC + EM cells, and the proliferation rate of UBA2-KD + mp53-OE cells was higher than that of UBA2-KD + EM cells. The levels of Bax, cleaved caspase-3, and cleaved caspase-9 were lower in NC + mp53-OE cells than in NC + EM cells and in UBA2-KD + mp53-OE cells than in UBA2-KD + EM cells (Fig. 5C). Moreover, the annexin V-FITC/PI apoptosis assay and PI staining showed that the proportion of apoptotic cells was lower in the NC + mp53-OE cell population than in that of NC + EM cells and in the UBA2-KD + mp53-OE cell population than in that of UBA2-KD + EM cells (Fig. 5D, E). Together, these results suggested that overexpression of the p53 mutant alleviated the effects of UBA2 knockdown on cell proliferation and apoptosis.

### Overexpression of c-Myc alleviated the effects of UBA2 knockdown on cell proliferation and apoptosis

**Fig. 6:**
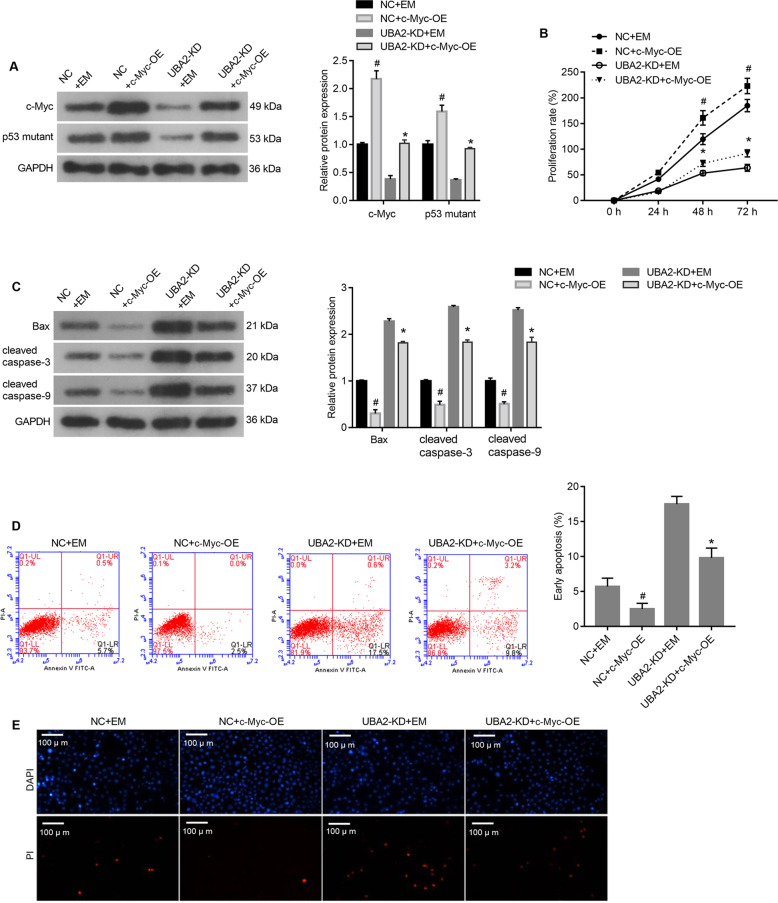
Alleviation of the effects of UBA2 knockdown on 786-O cell proliferation and apoptosis via overexpression of c-Myc. **A** Expression levels of the p53 mutant and c-Myc in cells transfected with c-Myc-overexpressing and control plasmids. **B** Proliferation rates of cells in (**A**). **C** Expression levels of proapoptotic proteins in cells in (**A**). **D** Proportions of apoptotic cells in the populations of cells in (**A**). **E** PI staining of cells in (**A**). ^#^*p* < .05 NC + c-Myc-OE vs. NC + EM; *p < 0.05 UBA2-KD + c-Myc vs. UBA2-KD + EM. DAPI 4′,6-diamidino-2-phenylindole, PI propidium iodide, OE overexpression, KD knockdown, NC negative control, mp53 p53 mutant, EM empty vector.

To examine whether UBA2 exerts its effects via a mechanism involving c-Myc, the c-Myc-OE plasmid was transfected into NC and UBA2-KD 786-O cells. NC and UBA2-KD cells transfected with the EM plasmid were used as controls. As shown in Fig. 6A, the expression levels of c-Myc and the p53 mutant were obviously higher in NC + c-Myc-OE cells than in NC + EM cells and in UBA2-KD + c-Myc-OE cells than in UBA2-KD + EM cells, indicating that the c-Myc-OE plasmid successfully overexpressed c-Myc and c-Myc overexpression affected the expression level of the p53 mutant.

Next, we evaluated whether overexpression of c-Myc alleviates the effects of UBA2 knockdown on cell proliferation and apoptosis. As shown in Fig. 6B, the proliferation rate of NC + c-Myc-OE cells was higher than that of NC + EM cells, and the proliferation rate of UBA2-KD + c-Myc-OE cells was higher than that of UBA2-KD + EM cells. The levels of Bax, cleaved caspase-3, and cleaved caspase-9 were lower in NC + c-Myc-OE cells than in NC + EM cells and in UBA2-KD + c-Myc-OE cells than in UBA2-KD + EM cells (Fig. 6C). Moreover, the annexin V-FITC/PI apoptosis assay and PI staining showed that the proportion of apoptotic cells was lower in the NC + c-Myc-OE cell population than in that of NC + EM cells and in the UBA2-KD + c-Myc-OE cell population than in that of UBA2-KD + EM cells (Fig. 6D, E). Together, these results suggested that overexpression of c-Myc alleviated the effects of UBA2 knockdown on cell proliferation and apoptosis.

## Discussion

We first found that UBA2 abundance was higher in ccRCC than in normal tissues, with consistent results obtained using three experimental methods, qRT-PCR, IHC, and western blotting. Although only 30 pairs of clinical specimens were included in this study, the results were consistent with those for 523 KIRC (another term for ccRCC [15] tissues and 100 normal tissues, analyzed in the GEPIA database. Moreover, the expression levels of UBA2 were significantly related to the tumor size, Fuhrman grade, and tumor stage. Therefore, the present study suggests that the UBA2 expression is abnormally high and may play an important regulatory role in ccRCC.

Promoting cell apoptosis is an effective way to prevent the malignant proliferation of cancer cells [16]. Thus, we focused on the effects of UBA2 on apoptosis by evaluating the apoptotic cell rate and the levels of the proapoptotic proteins Bax, cleaved caspase-3, and cleaved caspase-9. In vitro gain-of-function and loss-of-function assays showed that overexpression of UBA2 inhibited while UBA2 knockdown promoted cell apoptosis. These results were also confirmed in a nude mouse model of subcutaneous tumor formation. The in vitro and in vivo results suggested that UBA2 might play a role in suppressing apoptosis during ccRCC progression, and thus, inhibiting UBA2 expression may be a strategy to prevent the malignant proliferation of cancer cells. This suggestion was supported by the findings that UBA2 knockdown inhibited the proliferation of a ccRCC cell line, while UBA2 overexpression promoted cell proliferation. Moreover, our results are supported by those obtained by Kessler et al. [17], who showed that the depletion of UBA2/SAE2 significantly inhibited the growth of c-Myc-dependent SUM159 and MDA-MB-231 breast cancer cell lines.

To explore the underlying mechanisms, we investigated the effect of UBA2 on p53 expression. As a transcription factor, p53 can activate the expression of hundreds of genes [18]. These p53 target genes are directly involved in the regulation of the cell cycle, repair of DNA damage, and the regulation of cell senescence, differentiation, and apoptosis [19]. It is generally believed that wild-type p53 promotes the apoptosis of tumor cells [19, 20]. However, the function of p53 can be affected by some mutations [21]. There is a missense mutation, R248W (CGG → TGG), in codon 248 of exon 7 of the p53 gene in 786-O cells. In the present study, we found that overexpression of UBA2 could increase the level of the p53 mutant, and functional assays showed that overexpression of the p53 mutant inhibited apoptosis. These results suggested that the R248W mutation might affect the function of p53 and that UBA2 might play its role via the p53 mutant in the ccRCC cell line 786-O. Moreover, we found that overexpression of the p53 mutant alleviated the effects of UBA2 knockdown on cell proliferation and apoptosis, further indicating that UBA2 may play its role via the p53 mutant in the ccRCC cell line 786-O. Because there is no previous report on the role of the R248W p53 mutation in ccRCC and our results suggested that the R248W mutation affected the p53 function, we will further explore the role of this mutation in the ccRCC progression.

It has been reported that c-Myc is heavily SUMOylated and SUMOylation is required to support the oncogenic role of c-Myc [17]. Moreover, UBA2 depletion could suppress the growth of c-Myc-dependent cancer cells but had no significant effect on c-Myc-independent cancer cells [17]. These results suggested that UBA2 might play its role via a mechanism involving c-Myc. Therefore, we explored the relationship between UBA2 and c-Myc. Our results showed that UBA2 knockdown could promote apoptosis, inhibit cell proliferation, and decrease the c-Myc abundance, while overexpression of c-Myc alleviated the effects of UBA2 knockdown on cell proliferation and apoptosis. These results further indicated that UBA2 might play its role via c-Myc in the ccRCC cell line 786-O. It is generally assumed that c-Myc induces apoptosis [22] and is also an oncogenic transcription factor [23]. Apoptosis is the comprehensive outcome of the effects of both c-Myc and its target genes [22, 24, 25]. Thus, further studies are needed to explore the mechanistic aspects of the UBA2–c-Myc axis in apoptosis.

We also found that overexpression of UBA2 enhanced the levels of UBC9, PIAS1, and SENP1, which are the key enzymes of the SUMO modification system [26], indicating that UBA2 overexpression can activate the SUMO modification system. Although SUMOylation is similar to the ubiquitination process, SUMO-modified proteins are not degraded by proteasomes in the same way as ubiquitinated proteins but are more stable [26, 27]. The SUMOylation of p53 and c-Myc can enhance their stabilization [28, 29]. Based on our results showed that overexpression of UBA2 increased the levels of the p53 mutant and c-Myc, we suggest that UBA2 may regulate the expression of the p53 mutant and c-Myc via SUMOylation. In addition, we found that overexpression of the p53 mutant did not affect the c-Myc abundance, while overexpression of c-Myc increased that of the p53 mutant. These results suggested that c-Myc might play its role in apoptosis via a mechanism involving mutant p53. Besides the role in SUMO modification system, UBA2 also plays a role in regulating Wnt/β-catenin signaling pathway [8]. c-Myc is the downstream target protein of Wnt/β-catenin signaling pathway [30]. Therefore, UBA2 may regulate the expression of c-Myc through Wnt/β-catenin signaling pathway. In future experiments, we will focus on the molecular mechanism of UBA2 regulating c-Myc protein expression.

Our study has some limitations. First, although we found that UBA2 could affect the expression levels of the p53 mutant and c-Myc, the underlying mechanism was not further explored. Second, the mechanism of action of c-Myc in regulating the expression level of the p53 mutant was not investigated. Our future study will focus on these aspects.

In conclusion, this study explored the role and mechanism of UBA2 in ccRCC. It was shown that the expression of UBA2 was abnormally high in ccRCC. Meanwhile, UBA2 knockdown inhibited the proliferation and promoted apoptosis of ccRCC cells, whereas overexpression of UBA2 showed the opposite effects. Moreover, c-Myc and a p53 mutant played regulatory roles in these processes. Our study may provide a new theoretical basis for finding target molecules that can effectively promote cancer cell apoptosis or enhance proapoptotic effects of antitumor drugs.

### Supplementary information

a Reproducibility checklist

## Data Availability

All data generated or analyzed during this study are included in this article.
