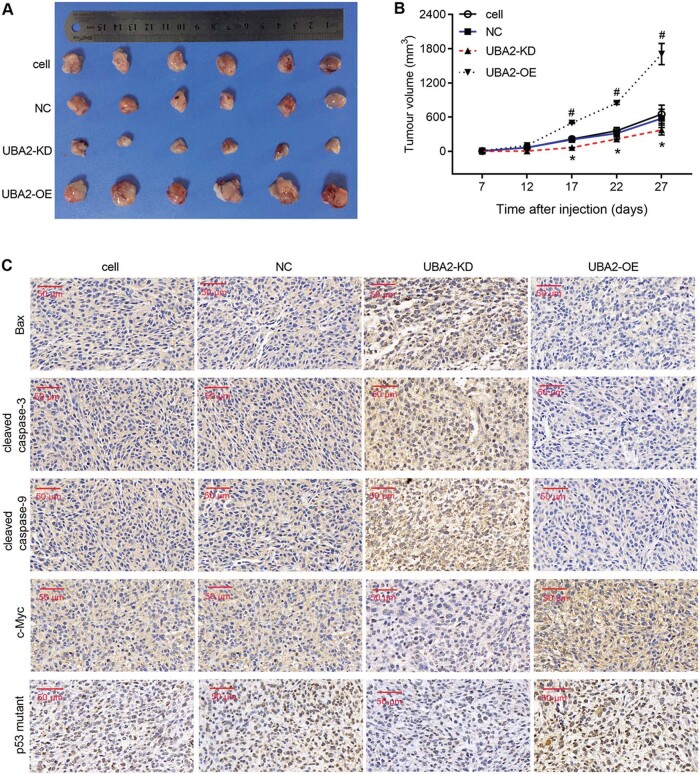# Correction: Knockdown of ubiquitin-like modifier-activating enzyme 2 promotes apoptosis of clear cell renal cell carcinoma cells

**DOI:** 10.1038/s41419-023-06384-w

**Published:** 2024-01-03

**Authors:** Guoxi Zhang, Junrong Zou, Jinglin Shi, Biao Qian, Kaiyang Qiu, Quanliang Liu, Tianpeng Xie, Zhihua He, Hui Xu, Yunfeng Liao, Yuting Wu, Yanmin Li, Guancheng Xiao, Yuanhu Yuan, Rihai Xiao, Gengqing Wu, Xiaofeng Zou

**Affiliations:** 1 Department of Urology, First Affiliated Hospital of Gannan Medical University, Ganzhou, Jiangxi 341000 China; 2 Department of Urology, Wan’an People’s Hospital, Ji’an, Jiangxi 343800 China

**Keywords:** Oncogenes, Oncogenesis

Correction to: *Cell Death and Disease* (2021) 12:1067 10.1038/s41419-021-04347-7, published online 09 November 2021

Since the publication of this paper, the authors have noted that there was an error Figure 4C. In that, the representative immunochemical staining image for c-myc of the NC group was misused. This error has now been rectified. The correct figure is shown below.
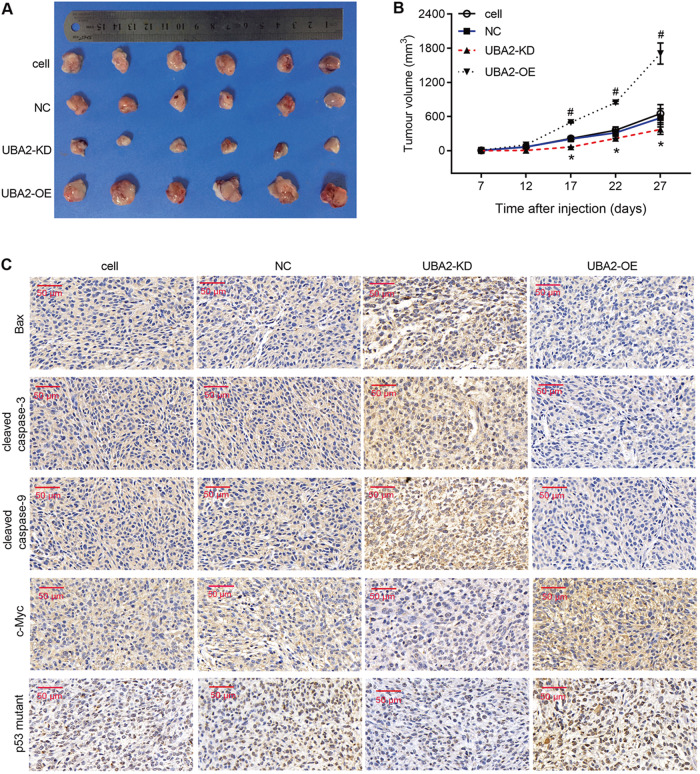


The incorrect figure is shown below.